# Targeted therapeutic options in early and metastatic NSCLC-overview

**DOI:** 10.3389/pore.2024.1611715

**Published:** 2024-03-28

**Authors:** Gabriella Gálffy, Éva Morócz, Réka Korompay, Réka Hécz, Réka Bujdosó, Rita Puskás, Tímea Lovas, Eszter Gáspár, Kamel Yahya, Péter Király, Zoltán Lohinai

**Affiliations:** Pulmonology Hospital Törökbálint, Törökbálint, Hungary

**Keywords:** NSCLC, driver oncogenes, targeted therapy, central nervous system efficacy, molecular testing

## Abstract

The complex therapeutic strategy of non-small cell lung cancer (NSCLC) has changed significantly in recent years. Disease-free survival increased significantly with immunotherapy and chemotherapy registered in perioperative treatments, as well as adjuvant registered immunotherapy and targeted therapy (osimertinib) in case of EGFR mutation. In oncogenic-addictive metastatic NSCLC, primarily in adenocarcinoma, the range of targeted therapies is expanding, with which the expected overall survival increases significantly, measured in years. By 2021, the FDA and EMA have approved targeted agents to inhibit EGFR activating mutations, T790 M resistance mutation, BRAF V600E mutation, ALK, ROS1, NTRK and RET fusion. In 2022, the range of authorized target therapies was expanded. With therapies that inhibit KRASG12C, EGFR exon 20, HER2 and MET. Until now, there was no registered targeted therapy for the KRAS mutations, which affect 30% of adenocarcinomas. Thus, the greatest expectation surrounded the inhibition of the KRAS G12C mutation, which occurs in ∼15% of NSCLC, mainly in smokers and is characterized by a poor prognosis. Sotorasib and adagrasib are approved as second-line agents after at least one prior course of chemotherapy and/or immunotherapy. Adagrasib in first-line combination with pembrolizumab immunotherapy proved more beneficial, especially in patients with high expression of PD-L1. In EGFR exon 20 insertion mutation of lung adenocarcinoma, amivantanab was registered for progression after platinum-based chemotherapy. Lung adenocarcinoma carries an EGFR exon 20, HER2 insertion mutation in 2%, for which the first targeted therapy is trastuzumab deruxtecan, in patients already treated with platinum-based chemotherapy. Two orally administered selective c-MET inhibitors, capmatinib and tepotinib, were also approved after chemotherapy in adenocarcinoma carrying MET exon 14 skipping mutations of about 3%. Incorporating reflex testing with next-generation sequencing (NGS) expands personalized therapies by identifying guideline-recommended molecular alterations.

## Introduction

**FIGURE 1:**
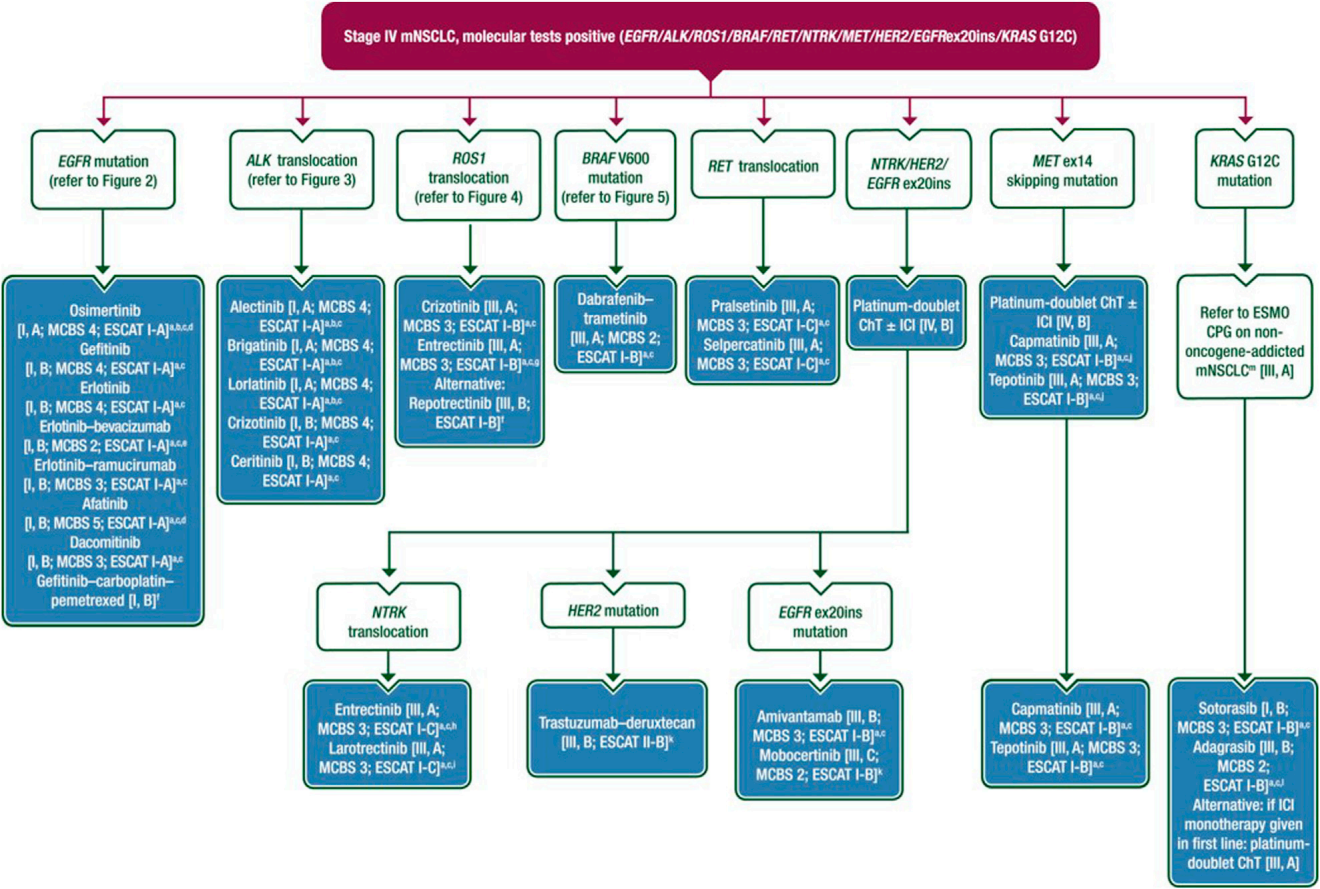
Treatment recommendation for stage IV mNSCLC based on the ESMO 2023 guideline [1].

In recent years, there has been an expansion in the treatment options of non-small cell lung cancer (NSCLC) with the advent of targeted therapies and immuno-oncology therapies [1, 2]. Lung tumors are heterogeneous, with distinct oncogenic drivers and tumor microenvironments. Tumor evolution results in distinct organ site metastases representing intratumor heterogeneity and the ongoing development of resistance mutations [3]. Recent advancements in cost-effective parallel high-throughput molecular diagnostics might drive personalized therapy beyond adenocarcinoma subtypes associated with the most targetable genetic alterations [3]. Prior to precision medicine, patients were treated uniformly without considering the differences in clinicopathological characteristics and genetic backgrounds of different patients. The careful selection for upfront treatments of brain metastases (BM) might be detrimental to outcomes [1, 2]. Now, it is clear that patients with oncogenic driver alteration-positive NSCLC are associated with better outcomes treated with frontline targeted therapy compared to chemotherapy and anti-Programmed death (anti-PD) immunotherapy [1, 2]. Today, the right choice of molecular diagnostics is increasingly important, following the careful classification of pathological diagnostics. While stand-alone single gene assays remain valid, increasing requirements for synchronous testing for multiple targets make massive parallel sequencing technology the preferred option [2]. DNA sequencing is the standard for mutation detection, and RNA sequencing is an emerging option for fusion gene detection. EGFR, ALK, and ROS1 alterations are common for young female non-smokers with adenocarcinoma [4]. While ALK and ROS1 mutations do not show ethnic prevalence, one can observe a four times elevated EGFR prevalence in the Asian population compared to the Western population [4]. KRAS and MET mutations occur in older age populations with smoker status and adenocarcinoma. There is no clear gender preference, but a distinct excess of KRAS mutations in the Caucasian population is observable. It is widely accepted that KRAS mutation in lung cancer is smoking-associated, but it is only proven for G12C [5, 6]. BRAF mutations occur in smokers without age or ethical tendencies. The BRAF V600 mutations are detected with a higher incidence for females; other BRAF mutations have a higher incidence for the opposite sex. HER2 mutations have a higher likelihood for female and never-smoker patients [4]. In contrast to the known associations of genetic alteration with clinicopathological characteristics, according to the latest guidelines, molecular testing is now the standard for advanced-stage adenoma-carcinoma-containing cancers independent of sex, ethnicity, or smoking [2]. Therefore, histology assessments are key in clinical practice, with a cautious interpretation of mixed histology specimens and NSCLC not otherwise specified (NSCLC NOS) because molecular testing is recommended for non-squamous NSCLC cases. Testing is also recommended for NSCLC cases below 50 years of age and all kinds of tobacco in patients who quit smoking more than 15 years ago, or in never (<100 cigarettes overall) or former light (≤15 6 pack-years) or long-time ex-smokers (quit >15 years ago). Importantly, the presence of any adenocarcinoma component in a biopsy specimen that is otherwise squamous should trigger molecular testing. Accordingly, cautious minimization of tissue slides used for immunohistochemistry (IHC) stainings and preserving material for molecular testing is critical. Oncogenic driver tests usually follow the Programmed death-ligand 1 (PD-L1) IHC testing for non-squamous cases. The present review summarizes the available data on targeted therapy strategies in treating NSCLC (Figure 1). A particular focus is given to central nervous system (CNS) activity that is detrimental in the era of better control of oligoprogressive disease. Additionally, for optimal treatment outcomes, we highlight the role of distinct molecular analyses based on accurate guideline-based histology classifications to avoid excluding patients from therapy. Nevertheless, the emergence of early-stage targeted therapies extends molecular testing beyond advanced-stage disease.

## Adjuvant osimertinib therapy in the treatment of NSCLC

As adjuvant treatment in NSCLC, osimertinib is the first targeted therapy approved based on the ADAURA trial. The ADAURA trial enrolled stage I/B -III/A patients with classical epidermal EGFR mutations (ex19del/L858R) who underwent complete tumor resection [7]. Patients were allowed to receive adjuvant chemotherapy before osimertinib, and optionally, they were allowed to start osimertinib therapy after surgery. According to the 1:1 randomization, the study group received osimertinib, and the other group received a placebo at a planned interval of 3 years. The primary endpoint was disease-free survival (DFS) in stage II/IIIA patients. DFS in the osimertinib group compared to placebo showed a significant benefit (hazard ratio (HR): 0.23, 95% CI 0.18–0.30) median DFS was 65.8 months for the osimertinib compared to 21.9 months for the placebo arm [8]. Subgroup analysis (gender, age, race, stage, mutation type) revealed significant benefits in DFS. Adjuvant osimertinib also showed efficacy without cytotoxic chemotherapy, with a significant benefit in DFS (HR: 0.23), and osimertinib given followed by adjuvant chemotherapy showed a similar significant benefit in DFS (HR: 0.16) [8].

## NSCLC with actionable EGFR mutations

The HER/Erb family epidermal growth factor receptor (EGFR) is a transmembrane tyrosine kinase (TK) receptor that stimulates cell proliferation, differentiation, survival, and motility through MAP kinase and PI3K signaling pathways [9]. Overexpression and increased activity of EGFR tyrosine kinase in non-small cell lung tumors were first described in 2004, which may result from mutation, deletion, or amplification of the tyrosine kinase coding region [10].

The mutation most commonly affects exons 18–21 and occurs in 10%–20% of the Caucasian population, predominantly in young, never-smoker women. In almost 90% of cases, a so-called “classical mutation” is encountered, a deletion of exon 19 or a point mutation of exon 21 (L858R). The exon 20 insertion is the third most common EGFR mutation, accounting for 4%–12% of all EGFR mutations, it is more common in women, non-smokers, and Asians and is associated with a worse prognosis. In addition to the exon 20 insertion mutation, the most common rare mutations include exon 18 G719X, exon 20 S768I and exon 21 L861Q, which occur in 1%–3% of cases and smoking history [11].

In locally advanced or metastatic non-small cell lung cancer, EGFR tyrosine kinase inhibitor (TKI) treatment is the recommended first-line therapy with confirmed actionable EGFR mutations. EGFR tyrosine kinase inhibitors are classified into three generations based on their appearance in chronological order. The first-generation includes the reversible binding agents gefitinib and erlotinib, the second-generation includes the irreversible ErbB/HER2 inhibitor afatinib and the EGFR/pan-HER inhibitor dacomitinib, while the third-generation is osimertinib. Since then, several studies have demonstrated the benefit of EGFR tyrosine kinase inhibitors in terms of tumor response, safety, quality of life, and progression-free survival (PFS) compared with conventional chemotherapy regimens [12].

## First-generation reversible EGFR inhibitors

Pioneering in the treatment of adenocarcinoma patients, gefitinib, in its pivotal phase 3 IPASS trial, showed a significant benefit in progression-free survival [median PFS (mPFS) gefitinib 9.5 months vs. chemotherapy 6.3 months; HR 0.48, 95% confidence interval (CI) 0.36–0.64 *p* < 0.001] and tumor response [objective response rate (ORR) 71.2% v 47.3%, *p* = 0.0001], with good quality of life maintained [13].

The efficacy of erlotinib was analyzed in the OPTIMAL trial in Asia and the EURTAC trials in Europe [14, 15]. A significant difference was demonstrated in favor of erlotinib in terms of overall survival (OS) and tumor response compared to the standard platinum-based chemotherapy regimens (EURTAC mPFS erlotinib 9.7 months vs. chemotherapy 5.2 months; *p* < 0.0001; ORR 64% vs. 18%; *p* < 0.0001) (OPTIMAL mPFS erlotinib 13.1 months vs. chemotherapy 4.6 months; *p* < 0.0001; ORR 83% vs. 36%; *p* < 0.0001) (5,6). Although no benefit in overall survival was demonstrated, following these trials, both products were registered in the first-line setting for treating EGFR mutation-positive stage IIIB/IV non-small cell lung cancer.

It is now well known that the first-generation drugs are most commonly associated with skin side effects (rash, xeroderma, pruritus, and paronychia), diarrhea, fatigue, and elevation of liver function, typically AST/ALT. The most common cutaneous side effects are acneiform rash, dry skin, itching, and nail bed lesions, which are well controlled by topical or systemic antibiotic treatment (doxycycline). Diarrhea can be reduced by per os medication (appropriate dose of loperamide), with drug dose reduction if necessary. Once the side effects are resolved, the original dose is often restored. Compared with chemotherapy regimens, first-generation regimens have shown a much better side-effect profile and fewer serious (grade 3–4) adverse events [13, 15–18].

A vascular endothelial growth factor (VEGF) inhibitor, Ramucirumab plus erlotinib, showed increased PFS compared to placebo plus erlotinib arm in patients with untreated EGFR-mutated metastatic NSCLC (mNSCLC) [19]. Safety was consistent with the safety profiles of the individual compounds in advanced lung cancer.

## Second-generation EGFR inhibitors

The second-generation EGFR tyrosine kinase inhibitors (afatinib and dacomitinib) are more potent EGFR and HER2 inhibitors, forming irreversible binding. The efficacy of afatinib was analyzed in LUX-lung studies. In the phase 2 LUX-lung 3 and LUX-lung 6 trials, afatinib showed significantly better tumor response and progression-free survival than platinum-based chemotherapy combinations (LUX-lung 3, mPFS for afatinib vs. chemotherapy, 13.6 months vs. 6.9 months; respectively, *p* = 0.0004; ORR, 56% vs. 23%; *p* = 0.001; respectively; LUX-lung 6, mPFS for afatinib vs. chemotherapy 11.0 months vs. 5.6 months; *p* < 0.0001; respectively, ORR 66.9% vs. 23%; *p* < 0.0001). [20, 21]. It should be noted that afatinib did not provide a benefit in OS in the overall patient group; however, in subgroup analyses, targeted therapy in patients with exon 19 deletion showed a significant benefit in overall survival (LUX-lung 3, OS for afatinib vs. chemotherapy 33.3 months 21.1 months respectively; LUX-lung 6, OS for afatinib vs. chemotherapy 31.4 months vs. 18.4 months, respectively). This benefit was not confirmed for point mutations [22]. Based on these studies, afatinib was registered as a first-line treatment for non-small cell lung tumors carrying EGFR mutations.

The LUX-lung 7 phase 2 trial comparing first- and second-generation EGFR tyrosine kinase inhibitors compared afatinib with gefitinib, where afatinib was shown to be superior in terms of progression-free survival (mPFS for afatinib vs. gefitinib, 11.0 months vs. 10.9 months; respectively, *p* = 0.017), however, the study did not show a significant benefit in terms of overall survival and more toxicity leading to dose reduction was observed when using the second-generation drugs [23]. A higher rate of G3-severe skin rash (9.4% vs. 3.1%) and G3 diarrhea (12.5% vs. 1.3%) was also observed in the afatinib group. Dacomtinib, also a second-generation irreversible EGFR and panHER inhibitor, was superior to gefitinib in first-line use in the ARCHER1050 phase 3 trial in terms of both PFS and OS [PFS for dacomitinib vs. gefitinib, 14.7 months vs. 9.2 months, respectively, *p* < 0.0001; median OS (mOS) dacomitinib vs. gefitinib, 34.1 months vs. 26.8 months, respectively], but again a less favorable side effect profile was observed with dacomitinib [24, 25].

## Third-generation EGFR inhibitors and T790 resistance mutation

Acquired resistance to first- and second-generation drugs develops after 9–13 months, with a T790 resistance mutation affecting exon 20 being confirmed in 50%–60% of cases [26]. This has led to the development of third-generation therapies [26]. Osimertinib is a third-generation irreversible EGFR TKI that is also effective in the presence of T790 resistance mutations. The efficacy of the first mutation selective TKI was analyzed in AURA trials. In the AURA 3 phase 3 trial, compared with platinum-based chemotherapy, osimertinib showed a significant PFS benefit in T790 resistance mutation-positive, locally advanced or metastatic NSCLC (mPFS osimertinib vs. chemotherapy, 10.1 month vs. 4.4 months, respectively, *p* < 0.001) [27]. This was followed by the phase 3 FLAURA trial, which compared the efficacy of osimertinib with first-generation agents; osimertinib achieved a significant benefit in both progression-free and overall survival, with osimertinib providing an 8.8 months OS benefit, reducing the risk of death by 20% (mOS osimertinib vs. first-generation EGFR inhibitor, 38.6 months vs. 31.8 months; respectively, HR 0.80; 95% CI, 0.6410 .997; *p* = 0.046). The side effect profile was similar, but G3 side effects were less frequent with osimertinib [28]. The most common osimertinib-induced adverse events were acneiform rash, diarrhea, and paronychia, and a small percentage of cardiomyopathy (∼1.4%–2.4%), QT prolongation (2.7%), and interstitial lung disease (ILD) (3.3%) were also described [29]. After 3 years of follow-up, 28% of patients received osimertinib, compared with only 9% in the comparator group. Notably, first-line osimertinib reduced the risk of CNS progression by 52% (HR, 0.48; 95% CI, 0.260.86; *p* = 0.014) and fewer *de novo* CNS metastases were recorded [30]. The importance of these results is demonstrated by the fact that 10%–30% of patients with NSCLC develop CNS metastases, which are associated with a worse prognosis and worse survival. EGFR mutations are present in 40%–60% of non-small cell lung tumors affecting the central nervous system, and the risk of central nervous system metastasis is higher in the presence of EGFR mutations, making the concentration of each drug in cerebrospinal fluid a key determinant. First- and second-generation drugs have limited brain penetration, while osimertinib rapidly crosses the blood-brain barrier (BBB) and reaches higher concentrations. Osimertinib, which has the best efficacy and most favorable side-effect profile of all EGFR inhibitors, is currently the drug for first-line treatment of EGFR mutant NSCLC, particularly in patients with BM [31]. If not available in the first line, first or second-generation agents should be administered, and in case of progression, efforts should be made to confirm T790 resistance mutation from liquid biopsy or repeated histological sampling. In the presence of a T790 resistance mutation, osimertinib is preferred while platinum-based chemotherapy is recommended in case of negative resistance mutation status. With the widespread use of osimertinib, the development of new resistance mutations is expected. Most commonly, mutation of exon 20 C797X, MET amplification, and HER 2 amplification have been described in addition to aberrations of other non-EGFR mediated pathways [1].

## Immunotherapy and targeted treatment options

The IMpower 150 phase 3 three-arm trial compared the combination of chemotherapeutic doublet immunotherapy and VEGF inhibitor with VEGF inhibitor-chemotherapeutic doublet and chemo-immunotherapy. EGFR mutant patients were also eligible for inclusion in the study. Subgroup analyses showed that a significant OS benefit was achieved with the combination of four regimens vs. VEGF inhibitor-chemotherapy doublet, regardless of the presence of EGFR mutations (EGFR positive subgroup mOS 26.1 months vs. 20.3 months; respectively), making the combination of four regimens an additional option after exhaustion of targeted therapies [32].

## NSCLC harboring a rare EGFR mutation

Approximately 10%–20% of non-small cell lung tumors carrying EGFR mutations carry rare EGFR mutations [33]. While the presence of classical activating mutations is a strong predictor of favorable tumor response to EGFR tyrosine kinase inhibitors, rare EGFR mutations, such as the exon 20 insertion mutation, are heterogeneous, largely resistant to first- and second-generation drugs, and the efficacy of third-generation osimertinib is limited. In recent years, several new products have been developed for this patient group. Mobocertinib is an irreversible EGFR and HER inhibitor targeting exon 20 alterations. In the phase 1/2 EXCLAIM trial, mobocertinib in the multilineage setting resulted in a 32% tumor response and a median progression-free survival of 7.3 months. Based on the phase 1/2 results, mobocertinib received Food and Drug Administration (FDA) approval. Amivantamab is a bispecific monoclonal antibody that prevents tumor growth and progression by blocking EGFR and c-MET pathways and stimulates immune-mediated destruction of EGFR and cMET-expressing cells. In the CHRYSALIS phase 1 study, patients with exon20 insertion mutations received multiple lines of amivantamab treatment, with an mPFS of 8.3 months and an ORR of 40%. Based on this study, the FDA approved amivantamab for the treatment of patients progressing on platinum-based chemotherapy. Although the use of amivantamab and mobocertinib has improved the tumor response and progression-free survival of this poor prognosis patient group, the presence of EGFR exon 20 insertions is still associated with poor prognosis and unfavorable survival, and further studies on drug development are needed [33].

In addition to the exon 20 insertion mutation, the most common rare mutations include exon 18 G719X, exon 20 S768I, and exon 21 L861Q, often in associated with other mutations [2]. Most clinical trials conducted to date have recruited patients with classical EGFR exon 19 deletion and exon 21 point mutations. An exception was the LUX-lung 2,3,6 trial (afatinib vs. chemotherapy), which included patients with rare EGFR mutations. Following a detailed, retrospective analysis of the trials, the benefit of afatinib in this patient group in terms of tumor response and PFS was published, leading to the registration of afatinib for the treatment of NSCLs with rare EGFR mutations. In the FLAURA 3 study comparing osimertinib and first-generation EGFR TKIs, rare EGFR mutation was an exclusion criterion, but in phase 2 Korean study (KCSG-LU15-09), patients with rare mutations showed a better tumor response and progression-free survival with osimertinib treatment, which was confirmed in some small patient studies. First-generation formulations have shown little activity in rare mutations. Although data are scarce, real-world studies to date suggest the use of second or third-generation drugs, afatinib or osimertinib, in the presence of a major EGFR rare mutation. The choice is a clinical decision, which is best made based on the side effects encountered, the patient’s general condition, and the products’ availability [34].

## Summary of EGFR targeted therapy

**FIGURE 2:**
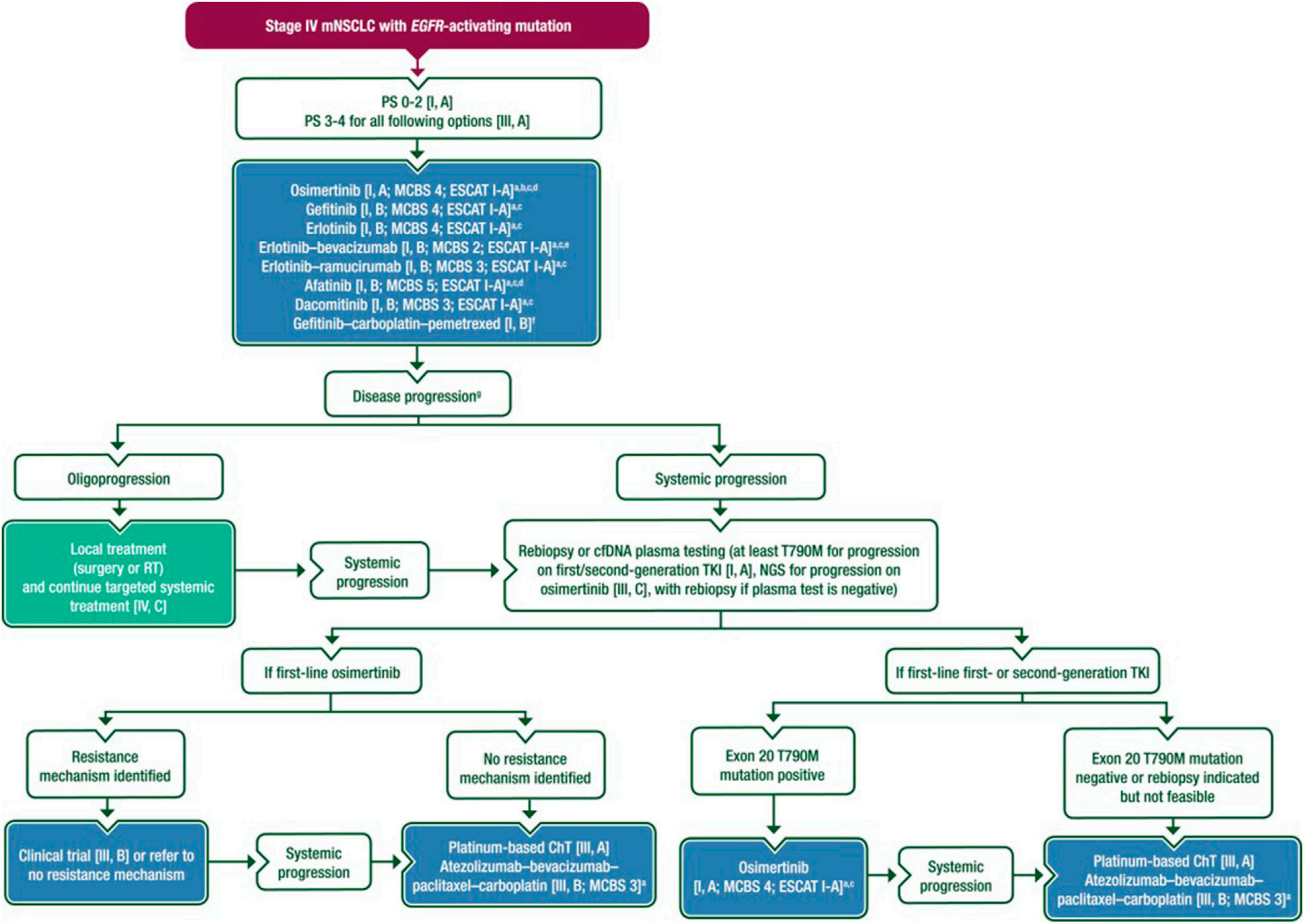
Treatment recommendation for EGFR mutant metastatic NSCLC based on the ESMO 2023 guideline [1].

Treatment recommendation for EGFR mutant metastatic NSCLC based on the ESMO 2023 guideline is shown in Figure 2. In all patients with advanced/metastatic lung tumors, molecular profiling is recommended after histological diagnosis. If an EGFR mutation is confirmed, first-line EGFR tyrosine kinase inhibitor therapy is recommended (I,A). When exon 19 del or exon 21 L858R EGFR mutation confirmed, osimertinib is the first choice, especially in the presence of BM (I, A). As first-line therapy, gefitinib chemotherapy and erlotinib-VEGF inhibitor therapy are also considered (I, B), but due to side effects and higher costs, EGFR TKI therapy alone is the preferred option (I, A). If osimertinib is not available, first-generation (gefitinib, erlotinib) or second-generation (afatnib, dacomitinib) agents are preferred (I, A). In case of progression, liquid biopsy or repeated tissue sampling is recommended to confirm T790 resistance mutation (I, A). In the case of a resistance mutation is present, second-line osimertinib is recommended (I, A), while platinum-based chemotherapy is the treatment of choice in case of negative results (III, A). In the case of osimertinib resistance, next-generation sequencing (NGS) is recommended to detect resistance genes (III, C). Enrolling in a clinical trial is a preferred option if available (III, B) (III, B). Otherwise, platinum-based chemotherapy is administered (III, A). After exhaustion of TKIs, chemotherapy doublet-immunotherapy-VEGF-quadruplet combination may be considered in patients with good overall Eastern Cooperative Oncology Group (ECOG PS) 0–1, if immunotherapy is not contraindicated (III, B) [1]. If oligoprogression is confirmed during EGFR tyrosine kinase inhibitor therapy, local metastasis treatment is recommended, while targeted therapy should be continued (III, A) [1]. In the presence of a rare mutation, non-exon 20 insertion, osimertinib, or afatinib is recommended (III, B). In the presence of exon20 insertion mutations, amivantamab can be given as second-line therapy in the case of progression after first-line therapy (III, B), while mobocertinib EMA approval is pending in these clinical settings (III, C) [1].

## Treatment of NSCLC with anaplastic lymphoma kinase (ALK) genetical alterations

ALK fusion genes are potent, albeit uncommon, driver oncogenes of non-small cell lung cancer. Notably, the detection of ALK fusion oncogene is of great importance because ALK-positive tumors are highly sensitive to ALK inhibitors, which significantly improves the life expectancy of patients.

## Diagnosis

Molecular testing for ALK fusion can be performed as part of standard clinical care in non-small cell lung cancer, primarily adenocarcinoma, from both tumor tissue and plasma samples [35]. Methods for detecting ALK translocation include NGS, IHC, Fluorescence *in situ* hybridization (FISH), RT-PCR. The standard methodology is FISH, but immunohistochemistry with monoclonal antibodies of high sensitivity and specificity and a validated method is an equivalent method for detecting ALK fusion oncoprotein. RNA-based multigene NGS assays are also suitable instead of IHC or FISH, with the advantage of simultaneous testing for other fusion oncogenes [36, 37].

## Epidemiology

The ALK fusion oncogene is present in 3%–5% of non-small cell lung cancers, with the majority of lung cancers carrying the gene being adenocarcinoma (97%). It is a disease of non-smokers or light smokers (<10 pack-years). Relative younger age at onset, with a median age of 52 years. The incidence of ALK molecular alteration in squamous cell carcinoma is limited. Cerebral metastasis is common, approximately 30% at the time of disease discovery [38, 39].

## First-line treatment of ALK-positive lung cancer with ALK tyrosine kinase inhibitor (ALK-TKI)

Crizotinib is a multitarget TKI, the first ALK inhibitor to improve the life expectancy of ALK-positive patients compared to chemotherapy in both first-line and subsequent-line settings.

In the phase 3 PROFILE 1014 trial, therapy-naive patients were included, and first-generation ALK inhibitor, crizotinib, was compared with pemetrexed and platinum doublet chemotherapy, and crossover was allowed [40]. At a median follow-up of 17 months, the primary endpoint, progression-free survival, was longer with crizotinib than with chemotherapy (median 10.9 vs. 7 months; HR 0.45, 95% CI 0.35–0.60). Objective tumor response was also increased (74% vs. 45%). At 46 months follow-up, there was no significant difference in overall survival (HR 0.76, 95% CI 0.55–1.05). However, after crossover adjustment, crizotinib also improved overall survival compared with chemotherapy (HR 0.35, 95% CI 0.08–0.72). The median overall survival of more than 4 years was reported in the crizotinib arm [40].

Ceritinib, a second-generation ALK inhibitor, has also been shown to be superior to chemotherapy when administered as first-line therapy [41].

The second-and third-generation ALK inhibitors are more effective than crizotinib in metastatic disease, including BM based on phase 1 randomized controlled trials, and are considered the preferred first-line agents. Second-generation ALK-TKIs are alectinib, brigatinib, and ensartinib (not approved by the European Medicines Agency (EMA)), and third-generation agents are lorlatinib [42–45].

**Alectinib** indicated for therapy naïve patients in first-line or previously treated with crizotinib in locally advanced or metastatic ALK-positive non-small cell lung cancer. In the phase 3 ALEX randomized controlled trial (N = 303), the median progression-free survival (mPFS) in the first-line setting compared with alectinib plus crizotinib was 35 months for alectinib and 11 months for crizotinib (HR 0.43, 95% CI 0.32–0.58). The results are not yet mature; the median overall survival (mOS) for the alectinib arm was not yet reached, and for the crizotinib arm, it was 57 months (HR 0.67 95% CI 0.46–0.98). Immature data showed a 5-year OS rate of 63% (alectinib) and 46% (crizotinib). Time to CNS progression was longer for alectinib than crizotinib (HR 0.16, 95% CI 0.10–0.28) in the overall population. The rates of grade 3–5 toxicity were similar for alectinib and crizotinib (52% vs. 56%). In the Alectinib arm, there was a higher incidence of anemia, myalgia, se bilirubin elevation, weight gain, and photosensitivity. Nausea, vomiting, and diarrhea were more frequent on the crizotinib arm [42, 46].

**Brigatinib** is currently approved for use in ALK-positive non-small cell lung cancer in locally advanced or metastatic ALK-positive patients previously treated with crizotinib or not previously treated with an ALK inhibitor. Brigatinib shows efficacy in a broad spectrum of ALK mutations. The phase 3 ALTA-1L randomized controlled trial (N = 275) compared brigatinib with crizotinib in ALK-TKI naive patients. Chemotherapy administration prior to randomization was not an exclusion criterion in the trial. In ALK inhibitor naïve ALK-positive patients at 3-year follow-up, the PFS was 43% vs. 19% for crizotinib vs. crizotinib arm, respectively, according to a standardized independent evaluation. mPFS at 9–11 months follow-up was 24 months versus 11 months (HR 0.48, 95% CI 0.35–0.66). The therapeutic benefit was observed in all subgroups and was prominent in patients with BM. The brain metastasis-related tumor response was significantly higher with brigatinib compared with crizotinib (78% versus 26%). mOS has not yet been reached by either group.

There was ILD/pneumonitis occurred in 4% of patients on brigatinib and 2% on crizotinib. The incidence of grade 3–4 ILD/pneumonitis was 3% versus 0.7%. The risk decreased by gradually increasing the dose of brigatinib (90 mg once daily for 7 days, then increased to 180 mg/day if tolerated). Symptoms associated with elevated creatine kinase (myalgia, muscle pain) did not differ significantly between the two agents. Nausea, diarrhea, constipation, peripheral edema, elevated liver function (GPT), and visual disturbances were more frequent with crizotinib. Grade ≥3 adverse events occurred in 61% with brigatinib and 55% with crizotinib [43].

**Lorlatinib** is recommended as monotherapy for adult patients with advanced ALK-positive non-small cell lung cancer who have not been previously treated with an ALK inhibitor. It is also for patients with advanced-stage ALK-positive NSCLC whose disease has progressed on first-line treatment with alectinib ceritinib or crizotinib.

In the phase 3 CROWN randomized controlled trial (N = 296), patients with the locally advanced or metastatic stage-naive disease were randomized to the lorlatinib or crizotinib arm. The mPFS was significantly better with lorlatinib than with crizotinib. In the first interim analysis, mPFS at 18 months of follow-up was not yet reached with lorlatinib versus 9.3 months with crizotinib (HR 0.28 95% CI 0.19–0.41). Lorlatinib showed robust CNS efficacy. Grade 3–4 adverse events occurred in 72% of patients treated with lorlatinib and 56% with crizotinib. Hypercholesterolaemia and hypertriglyceridemia occurred in >70% of patients on lorlatinib and neurocognitive side effects may affect the first-line use of lorlatinib [45, 47, 48].

## Duration of treatment

Treatment with ALK inhibitors is continued until disease progression. In the case of oligoprogression, local intervention is recommended in addition to the continuation of ALK-TKI. A more potent next-generation ALK inhibitor or standard chemotherapy is indicated for extensive progressive disease.

## Treatment for progression on crizotinib

For progression following crizotinib, alectinib or brigatinib is recommended, given their systemic and CNS efficacy and good tolerability.

Alectinib—In the phase 3 ALUR study (N = 107), patients with advanced ALK-positive disease pretreated with platinum-based chemotherapy and crizotinib were randomized to alectinib or mono-chemotherapy (pemetrexed or docetaxel). PFS was longer with alectinib, 7.1 months vs. 1.6 months (HR 0.32, 95% CI 0.17–0.59), and the number of grades ≥3 adverse events was lower with alectinib (27% versus 41%). CNS efficacy was also better with alectinib [49].

Brigatinib—In the phase 2 ALTA study (N = 222), patients refractory to crizotinib at 1 × 90 mg/day (arm A) or 1 × 180 mg/day (arm B) after a seven-day 1 × 90 mg/day lead-in period with brigatinib had an mPFS of 9.2 months versus 16.7 months at lower and higher doses of the agent, respectively. The median overall survival (OS) was 29.5 months versus 34.1 months. In patients with baseline BM, the independently assessed CNS objective tumor response was 50% versus 67%. Both arms had low rates of grade ≥3 toxicity [50].

**Ceritinib** is not preferable because it is less effective than the former in cross-trial comparisons. In the open-label ASCEND-5 study, 231 patients were randomized to ceritinib 750 mg/day or chemotherapy arm after crizotinib treatment, with ceritinib having better PFS (5.4 versus 1.6 months; HR 0.49) and ORR (39.1% versus 6.9%), both statistically significant. Nevertheless, OS analysis is still immature. Due to crossover, the OS advantage is expected to be decreased in the ceritinib arm. While initial studies used a ceritinib dose of 750 mg/day with fasting intake, a randomized open-label trial found an equivalent dose of 450 mg/day with meals was associated with lower gastrointestinal toxicity [51].

Although a phase 2 trial has shown lorlatinib to be effective in progression on crizotinib (ORR 69%, intracranial ORR 68%, mPFS not yet achieved), the EMA prescribing after crizotinib requires the prior use of a second-generation TKI [48, 52].

## Treatment for progression on second-generation ALK TKI

**Lorlatinib** is a third generation ALK-TKI. Lorlatinib is effective against acquired resistance mutations in most ALK kinase domains, including G1202R and other ALK kinase domain mutations. Lorlatinib is the preferred agent for alectinib-induced resistance [53]. This is probably also true for other second-generation ALK inhibitors [52]. Lorlatinib is also characterized by high CNS penetration.

In a phase 2 trial, lorlatinib in patients previously treated with one or more ALK inhibitors resulted in high objective tumor response (47%), complete remission (2%) and partial tumor response (45%). Following crizotinib, treatments with lorlatinib, the ORR was 73%, and mPFS was 11.1 months. After one or more second-generation ALK inhibitors, ORR was 40% and mPFS was 6.9 months. At >30 months median follow-up, mOS was 21 months. The most common adverse events in this study were hypercholesterolemia (81%), hypertriglyceridemia (61%), edema (43%) and peripheral neuropathy (30%). Serious treatment-related adverse events developed in 7% of patients, the most common being cognitive impairment (1%). In patients who progressed with second generation ALK-TKI, the ORR for lorlatinib was higher when an ALK mutation was present in addition to the ALK fusion oncogene, suggesting that second generation ALK mutations may be associated with the development of a new oncogene. Therefore, genotyping ALK mutations after progression on second generation ALK inhibitors may identify patients more likely to benefit from lorlatinib treatment [52].

Although lorlatinib has not been studied in comparison with chemotherapy in alectinib-resistant disease, lorlatinib after alectinib is recommended because chemotherapy can only achieve poorer survival after progression from ALK-TKI.

**Alternative target therapies** -ceritinib and brigatinib also show activity in progression after alectinib based on small observational studies [54, 55].

## Treatment options for subsequent lines in ALK-positive NSCLC

The IMpower 150 trial suggests chemo-immunotherapy + VEGF inhibitor, the median PFS in patients with EGFR activating mutation + ALK-positive subgroup in TKI pretreated patients were 9.7 vs. 6.1 months; respectively, (HR 0.59, 95% CI 0.37–0.94). Nevertheless, there is no evidence on the efficacy of mono-immunotherapy in the presence of ALK-positivity. Chemotherapy alone is an acceptable additional option with moderate activity in this patient population. Some experts recommend a standard combination of pemetrexed, carboplatin, and pembrolizumab combinations for lung adenocarcinoma, although clinical trials demonstrating the efficacy of this combination have excluded ALK-positive patients [56, 57].

## Treatment of brain metastases in ALK-positive NSCLC

In cases of BM, both for symptomatic or asymptomatic cases, second or third generation ALK-TKI is recommended, as these agents have good blood barrier penetration and CNS efficacy. The majority of patients with BM, whether TKI naïve or treated with crizotinib, are likely to respond to these agents and may be able to defer surgical intervention or radiotherapy, thereby reducing morbidity associated with local care. However, surgical treatment may be considered as initial therapy in cases of spatial disproportionation or risk of herniation due to massive BM [58].

## Summary of ALK targeted therapy

**FIGURE 3:**
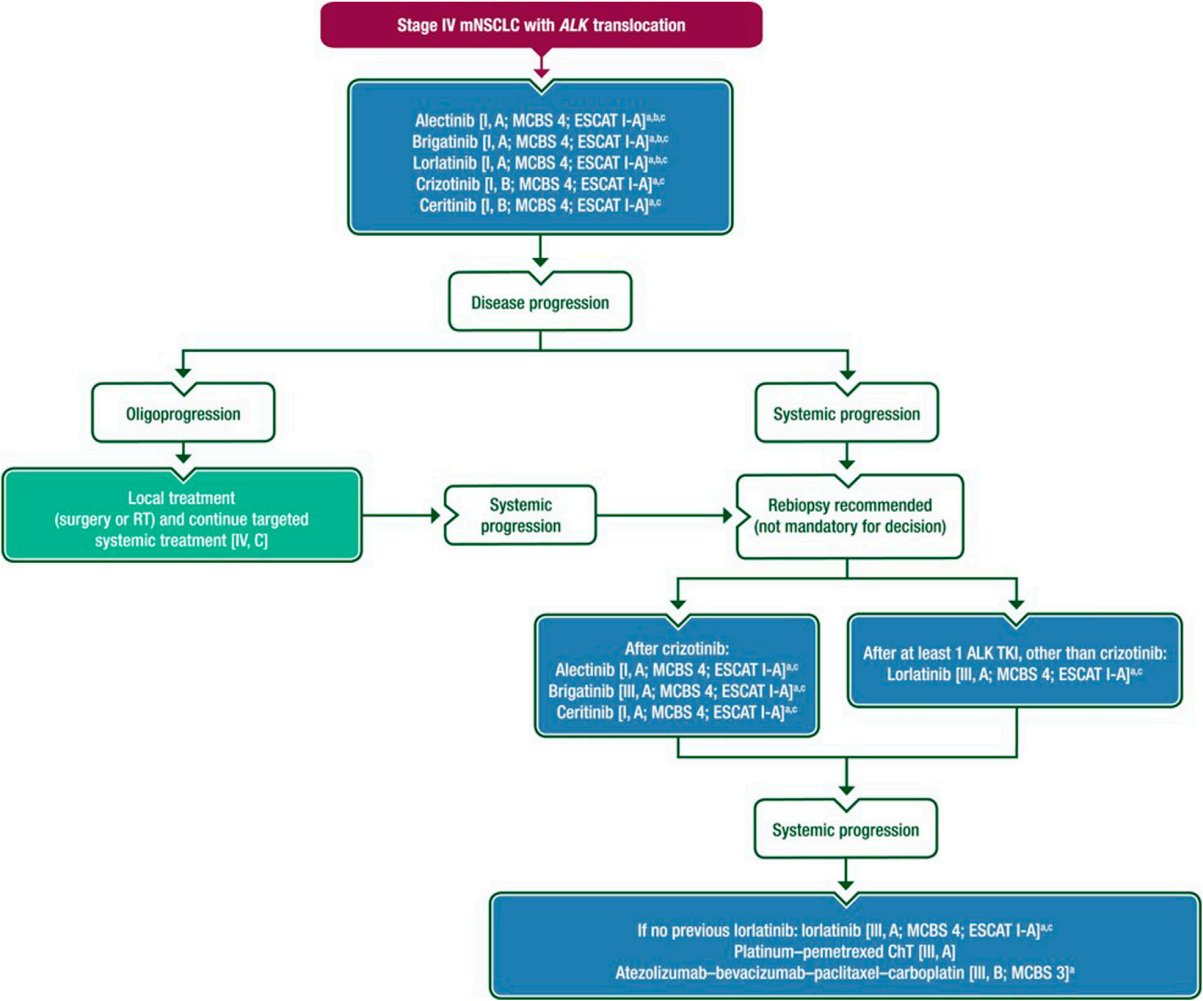
Treatment recommendation for ALK translocated metastatic NSCLC based on the ESMO 2023 guideline [1].

Treatment recommendation for ALK translocated metastatic NSCLC based on the ESMO 2023 guideline is shown in Figure 3. NSCLC with ALK rearrangements is a subtype of lung cancer with specific clinical and pathological features. Due to the availability of effective therapies, all lung adenocarcinomas should be investigated for ALK fusion oncogenicity. In locally-advanced or metastatic stage ALK-positive NSCLC, a second or third-generation ALK inhibitor is recommended in the frontline setting. Treatment should be continued until progression or intolerable toxicity. In case of oligoprogression in mildly symptomatic or asymptomatic patients, local ablative therapy for the progressive formulation is recommended with continuation of the ALK inhibitor. For progression on a second-generation ALK inhibitor, lorlatinib is recommended over chemotherapy or other ALK inhibitors. Following the exhaustion of TKIs, platinum doublet CHT or in CHT combination with bevacizumab and/or anti-PD immunotherapy can be administered if the patient is still fit for further treatment.

Further studies are needed for TKI-treated ALK-positive cases to determine whether identifying specific tyrosine kinase domain mutation can identify appropriate next steps in therapy. Nevertheless, some preliminary data suggest that specific kinase domain mutations may impact the following line of therapy [2]. Broad genomic profiling may be the most informative approach to examining potential resistance mechanisms, which may require repeated sampling during treatments. Assay methodology selection can impact the ability to identify subclonal events in this setting.

## KRAS mutant NSCLC treatment

KRAS mutations activate several additional signaling pathways, occur in about 20%–25% of lung adenocarcinomas and are usually associated with lung cancer in smokers [59]. G12G KRAS mutation subtype is associated with smoking status [6].

The presence of the KRAS mutation in early lung cancer does not seem to affect overall survival, however others have shown that it is associated with a poor prognosis [60]. The focus of targeted therapy for KRAS mutant lung cancer is on irreversible inhibitors of KRAS G12C. KRAS G12C mutations account for nearly 50% of all KRAS mutations [60].

## Treatment of non-small cell lung cancer with KRAS G12C mutation

### First line treatment

First-line therapy regimens are recommended similarly to non-oncogene-dependent, non-squamous NSCLC [1].

### Second-line treatment

Targeted treatment for KRAS G12C mutant tumors after first-line platinum-based chemotherapy and/or anti-PD immunotherapy is considered.

Sotorasib is the first target agent to receive regulatory approval for KRAS G12C mutant locally advanced or metastatic adenocarcinoma in patients who have received at least one prior systemic therapy [61].

In the randomized, open-label, phase 3 CodeBreak 200 trial (N = 345), patients with KRAS G12C mutations were randomized to sotorasib or docetaxel after progression on platinum-based chemotherapy and anti-PD immunotherapy treatment. Better PFS was achieved with sotorasib than docetaxel based on independent unblinded assessment (5.6 versus 4.5 months HR 0.66, 95% CI 0.51–0.86), with fewer grade ≥3 toxicities (33 versus 40%) and fewer serious adverse events (11 versus 23%). Overall survival was similar in the two groups (10.6 months with sotorasib and 11.3 months with docetaxel, HR 1.0). The most common grade ≥3 treatment-related adverse events were diarrhea (12%) and elevated transaminase levels (5%–8%) [62].

In the phase 1 CodeBreak 100 trial, sotorasib achieved an objective tumor response of 41%, mPFS of 6.3 months, OS of 12.5 months, and a two-year survival rate of 33% [63].

Several drug interactions are known to occur with sotorasib. It is not recommended for co-administration with antacids such as proton pump inhibitors, H2 receptor blockers, potent cytochrome P450 3A4 (CYP3A4) inducers, and certain CYP3A4 and P-gp substrates [61].

Adagrasib has been granted conditional marketing authorization by the EMA to treat advanced non-small cell lung cancer with KRAS G12C mutation and progression on at least one prior systemic therapy [64].

In the Krystal-1 single-arm, phase 1–2 study (N = 116), KRAS G12C mutant patients received 2 × 600 mg of adjuvant adagrasib daily after prophylactic chemotherapy and PDL1 inhibitor immunotherapy. The mPFS was 6.5 months, objective tumor response was 43%, the median duration of response was 8.5 months, and OS was 12.6 months. In 33 patients with previously treated stable BM, the intracranial confirmed objective response was 33%. Grade ≥3 treatment-related adverse events occurred in 45% of patients, the most common being fatigue, nausea and elevated liver function tests. Two grade 5 events occurred: heart failure in a patient previously known to have pericardial effusion and one pulmonary hemorrhage [65].

### Summary of targeted therapy in KRAS mutant NSCLC

For patients with KRAS G12C mutant NSCLC progressed after a prior line of therapy, second-line sotorasib or adagrasib may be recommended over subsequent chemo and/or immunotherapy.

### Treatment of NSCLC with ROS1 genetical alterations

**FIGURE 4:**
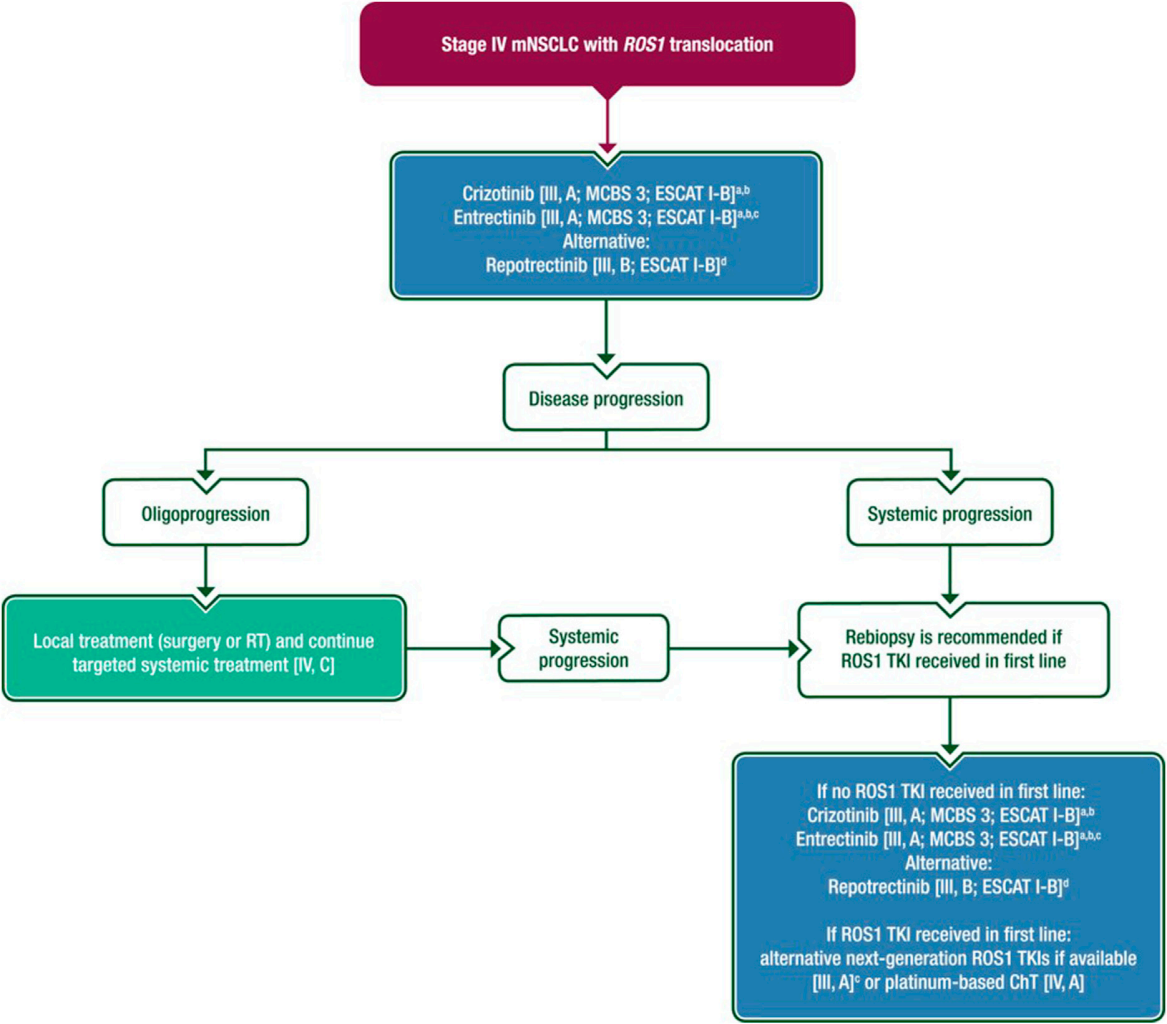
Treatment recommendation for ROS1 translocated metastatic NSCLCbased on the ESMO 2023 guideline [1].

The ROS1 proto-oncogene encodes a tyrosine kinase of the insulin receptor family, which is structurally similar to ALK. ROS1 gene fusion was first identified in 1987 in the glioblastoma cell line U118MG [66]. Since then, ROS1 gene rearrangements have been observed in 22 adult and pediatric malignancies [67]. It is detectable in 1%–2% of NSCLC, with a higher prevalence in non-smoking, younger women. ROS1 mutations do not co-occur with other driver mutations, with rare exceptions including in *EGFR* (1/166) and *KRAS* (3/166) and no co-occurring *ROS1* and *ALK* alterations [68]. They are almost exclusively detected in adenocarcinoma, but rare cases are also found in squamous cell, pleiomorphic, and large cell lung carcinoma [69]. FISH is the gold standard method for the detection of ROS1 gene rearrangements. IHC has high sensitivity but low specificity and is not recommended as a primary determinant for treatment. In the case of a positive or inconclusive ROS1 IHC result, confirmatory FISH, NGS, and RT-qPCR should be performed [69, 70]. Treatment recommendation for ROS1 translocated metastatic NSCLC based on the ESMO 2023 guideline is shown in Figure 4.

**Crizotinib** was the first TKI inhibitor to be approved by both the EMA and the FDA for the treatment of ROS1 mutant non-small cell lung cancer based on the results of the phase 1 PROFILE 1001 clinical trial [71]. The study cohort included 53 patients with ROS1-positive NSCLC, most of whom (87%) had previously received first-line platinum-based chemotherapy. The ORR for crizotinib was 72%, associated with a disease control rate (DCR) of 90%. The mPFS and mOS were 19.3 and 51.4 months, respectively. The median duration of therapy with treatment was 22.4 months (15.0–35.9 months) [71]. Subsequently, several retrospective and prospective phase 2 studies have demonstrated the efficacy of crizotinib [69]. However, it is important to emphasize that the drug has a low BBB penetration and, due to the poor brain penetration with crizotinib, the primary site of progression is the central nervous system [72].

**Entrectinib** is a new generation TKI that inhibits tropomyosin-related kinases in addition to its anti-ROS1 activity. Based on the results of an analysis of data from three prospective phase 1 and 2 clinical trials (ALKA-372–001, STARTRK-1, STARTRK-2), the ORR with entrectinib was 67% and mPFS was 15.7 months. A significant proportion of the 161 patients included in the study (62.7%) had received prior systemic therapy and 34.8% had BM at baseline. The 24 patients who had measurable BM at diagnosis had an intracranial ORR of 79%, mPFS of 12 months and mOS of 26.3 months. The majority of adverse events associated with entrectinib were grade 1 and 2, and overall, the tolerability and safety profile of the agent was similar to other ROS1 inhibitors [73, 74]. Based on these results, entrectinib was granted a marketing authorization by the FDA in 2019 and by the EMA in 2020. Entrectinib is the first line drug for known BM based on the ESMO 2023 recommendation [1].

**Ceritinib** is a second-generation ALK/ROS1 TKI with significant central nervous system activity. In a phase 2 clinical trial in Korea, 32 patients with advanced ROS1-positive disease, mostly crizotinib-naive (n = 30), were treated with ceritinib. In the whole cohort, ORR was 62%, mPFS was 9.3 months, and DCR was 81%. Among patients who had not received crizotinib treatment, ORR reached 67% and mPFS 18.3 months. Of note, no treatment response was observed in the two patients previously treated with crizotinib while on ceritinib [75]. Based on these results, ceritinib may be considered for crizotinib treatment in patients with ROS1-positive NSCLC who have not previously received crizotinib; however, currently, the agent is neither FDA nor EMA-approved.

**Lorlatinib** is a third-generation ALK/ROS1 TKI that penetrates the brain and has been effective in a phase 1 and phase 2 single-arm clinical trial that enrolled 69 ROS1-positive patients [52, 76]. The ORR in the TKI-naïve cohort (n = 21) was 62%, mPFS 21 months, and intracranial ORR 64%, compared to a group of previously crizotinib-treated patients (n = 40), where ORR was only 35%, mPFS 8.5 months, and intracranial ORR 50%. There was also a significant difference in the median duration of response (mDOR) (25.3 months vs. 13.8 months). Along with ceritinib, lorlatinib does not have FDA or EMA approval.

**Repotrectinib** is a new generation ROS1/TRK/ALK tyrosine kinase inhibitor. In the phase 1/2 TRIDENT-1 clinical trial in the ROS1 TKI-naïve group (n = 71), ORR was 79% and mDOR was 34.1 months [77, 78]. In patients who had previously received ROS1 TKI therapy but did not receive chemotherapy/immunotherapy (n = 56), ORR was 38% and mDOR was 14.8 months. Based on these results, in November 2023, the FDA approved repotrectinib to treat ROS1-positive NSCLC [79].

**Brigatinib, cabozantinib, thaletrectinib, and ensartinib** also have ROS1 inhibitory effects based on preclinical and phase 1/2 studies [80–83].

### RET-positive NSCLC treatment

The RET gene encodes a tyrosine kinase-activated membrane receptor protein, primarily involved in the differentiation of the enteric nervous system and urogenital tract [84]. Oncogenic RET alterations can be detected in several solid tumor types [85], such as thyroid cancers, NSCLCs, pancreatic, colorectal, and breast tumors, and are involved in, among others, multiple endocrine neoplasia type 2 [86], and Hirschprung’s disease [87]. RET gene rearrangement is detected in 1%–2% of NSCLCs; these tumors are typically found in non-smoking, younger patients and are associated with an increased risk of BM [88]. Histologically, they are almost exclusively of the adenocarcinoma subtype; others showed 92.3% non-squamous histology [89]. Several fusion partners of RET are known, the most common being KIF5B and CCD6C [90]. IHC and RT-PCR have proven to be unreliable methods for diagnosing RET-positive NSCLCs due to their low sensitivity and variable specificity and are replaced by FISH and NGS [85, 90].

Selpercatinib is a low molecular weight drug that can penetrate the BBB and is a highly selective RET tyrosine kinase inhibitor (TKI) that has demonstrated efficacy in RET translocation-positive NSCLC in the LIBRETTO-001 phase 1/2 clinical trial [91]. The trial enrolled 105 patients previously treated with platinum-based chemotherapy and 39 therapy-naive patients. For pretreated patients, the ORR was 64% (95% CI, 54%–73%) and the mDOR was 17.5 months. In the therapy-naïve group, the ORR was 85% (95% CI, 70%–94%). Of note, 91% of the n = 11 patients with BM observed an intracranial clinical response. The most common grade 3 or more severe adverse events were hypertension (14%), elevated alanine aminotransferase (12%) and aspartate aminotransferase (10%) levels, hyponatremia (6%) and lymphopenia (6%). Based on these results, both FDA (2020) and EMA (2021) have approved selpercatinib for the treatment of locally advanced and metastatic NSCLC [92]. The randomized phase 3 multicentre trial (LIBRETTO-431) compared the efficacy of first-line selpercatinib with or without chemotherapy (carboplatin/cisplatin + pemetrexed) with or without pembrolizumab [93]. The results of the study were presented at the ESMO Congress 2023 [94, 95]. The selpercatinib group had a significantly higher mPFS compared to the chemotherapy ± immunotherapy group (24.8 months vs. 11.2 months; HR:0.465, CI: 0.309–0.699; *p* < 0.001) [95].

**Pralsetinib** is the other selective RET inhibitor that will be registered by the FDA in 2020 and by the EMA in 2021, based on the results of the ARROW clinical trial. However, while the EMA approval (for both selprecatinib and pralsetinib) is only valid for patients who have not previously received RET TKI therapy, the FDA approval does not include such a restriction [96–99]. The ORR was 72% in the treatment-naïve (n = 75) and 59% in the group of patients (n = 136) who had received prior platinum-based chemotherapy. The mDOR was not reached in the therapy-naïve group and 22.3 months in the pretreated group. As with selpercatinib, pralsetinib has significant intracranial activity, with an intracranial ORR of 70% (95% CI, 35%–93%) in the group of patients with BM (n = 10, all of whom had received prior chemotherapy) in the study. The agent’s tolerability and side effect profile were similar to the other TKIs [100]. In the ongoing AcceleRET phase 3 clinical trial, similar to the LIBRETTO-431 trial, first-line pralsetinib therapy is being compared with platinum-based chemotherapy ± pembrolizumab [101, 102]. Results of the trial are expected in 2024.

### BRAF mutant NSCLC treatment

Mutations in BRAF (V-raf murine sarcoma viral oncogene homolog B) are mutations in the MAPK mitogen-activated protein kinase pathway, which affects downstream signaling proteins. BRAF mutations are alternative oncogenic drivers in NSCLC, which mutually exclude EGFR mutations and ALK and ROS1 rearrangements. The incidence of lung adenocarcinoma is 4.5% [103]. BRAF mutations in the serine/threonine kinase domain most commonly affect V600 [104]. Kinase inhibitors are now available for BRAF V600E mutations. These include dabrafenib, a serine/threonine kinase inhibitor, and trametinib, which has both serine/threonine and tyrosine kinase inhibitory activity [105].

The registration of the drugs was based on a prospective, multicentre, multicohort phase 2 study (BRF113928). The study enrolled 171 patients with metastatic NSCLC with BRAF-V600E mutations, of whom 78 patients received dabrafenib monotherapy (Cohort A), 57 patients received the MEK inhibitor trametinib in combination in multiple lines (Cohort B) and 36 patients received first-line combination therapy (Cohort C). The dose of dabrafenib was 2 × 150 mg/day in both monotherapy and combination therapy, while trametinib treatment was administered at 1 × 2 mg/day. With dabrafenib monotherapy, the response rate (ORR) was 33%, the mPFS was 5.5 months, and the mDOR was 9.6 months. In pretreated patients with the dabrafenib-trametinib combination, the ORR was 68%, mPFS was 10.2 months, and mDOR was 9.8 months. In previously untreated patients on dabrafenib-trametinib combination therapy, the ORR was 64%, mPFS was 10.8 months, and mDOR was 10.2 months. In patients receiving pretreated combination therapy (Cohort B), a median overall survival (OS) of 18.2 months was observed, with 4-year and 5-year survival rates of 34% and 22%, respectively, representing a significant improvement compared to both dabrafenib monotherapy and conventional chemotherapy. The combination of dabrafenib and trametinib is indicated for the treatment of patients with advanced metastatic-stage NSCLC with BRAF V600E mutations (the study on which the registry was based included only BRAF V600 mutation-positive patients, so its efficacy in wild-type BRAF mutant NSCLC has not been proven) [106].

NSCLC with distant metastases should be tested for BRAF V600 mutation status [ESMO II, A]. For NSCLC with BRAF V600E mutation in metastatic stage, first-line treatment with dabrafenib + trametinib is recommended [ESMO III, A; ESCAT: I-B]. If patients have received first-line BRAF and MEK inhibition, platinum-based chemotherapy with or without immunotherapy may be recommended as second-line treatment [ESMO IV, B] [106].

### MET exon 14 skipping mutation and MET amplification in NSCLC

Oncogenic activation of the MET (mesenchymal-epithelial transition) signaling pathway can be caused by overexpression, gene amplification, gene rearrangements, and various mutations [1].

MET exon 14 skipping mutations are found in about 3%–4% of younger/smoker/gender patients with NSCLC, mostly in cases where no other driver mutation can be identified, and more often in elderly and smoker patients. In addition to adenocarcinoma, its occurrence has also been observed in sarcomatoid carcinoma. It is considered an unfavorable prognostic marker. The skipping mutation can be detected by DNA- or RNA-based NGS (ESMO IB), while MET amplification can be detected by immunohistochemistry or *in situ* hybridization (ESMO IIB) [107–109].

Detection of MET overexpression is not associated with effective target therapy, but MET exon 14 skipping mutations or MET amplification is associated with the efficacy of MET inhibitors. MET amplification occurs in 1%–6% of NSCLC cases and may be a cause of acquired resistance to EGFR and ALK inhibitors. For MET amplification, the method of detection and the definition of high gene copy numberstill need to be standardized, while for high gene amplification the MET inhibitor capmatinib has been shown to be effective, but the FDA and EMA have not yet approved its use in this indication. In the case of MET exon 14 skipping mutations, registered targeted treatment options are available. Detection of MET exon 14 skipping mutation and MET amplification is recommended in the initial evaluation of patients diagnosed with non-squamous NSCLC (ESMO IIA).

The FDA has approved capmatinib and tepotinib for the first-line treatment of NSCLC with MET exon 14 skipping mutations, but the EMA has not approved it for first-line, only second- and multiline therapy at present. Thus, first-line platinum-based chemotherapy ± immunotherapy is recommended in these cases.

For patients with MET-amplification, platinum-based chemotherapy with/without immunotherapy is recommended as first-line treatment (ESMO IVB). Following first-line treatment, treatment with capmatinib or tepotinib monotherapy is recommended for patients with MET exon 14 skipping mutation-positive NSCLC (ESMO III A). For tumors carrying less frequent driver mutations, there is little data on the efficacy of immunotherapy, and in these cases, platinum-based chemotherapy or chemotherapy + immunotherapy is recommended if targeted therapy is not an option, while monotherapy immunotherapy is not recommended [108].

### Capmatinib

Capmatinib is a potent, selective MET receptor inhibitor and has been shown to be effective in various types of MET activation *in vitro* and *in vivo* tumor models [110]. Capmatinib can cross the BBB.

The registration of the medicine was based on the results of the GEOMETRY mono-1 study [110, 111]. The phase 2, open-label, multi-arm study enrolled 364 patients with advanced (stage IIIB or IV) NSCLC who were found to have MET amplification or MET exon 14 skipping mutations. Patients who had received prior chemotherapy and subjects who had not yet received treatment were also included. Patients received capmatinib therapy in the form of 2 × 1,400 mg tablets daily.

In patients with MET exon 14 skipping mutations, the ORR was 41% in previously treated patients and 68% in previously untreated patients. The mPFS was 5.4 months in previously treated subjects and 12.4 months in patients who received first-line treatment. The effect of capmatinib treatment was typically rapid, with the vast majority of patients showing a response (82% of the previously treated patients and 68% of those who had not received treatment previously) having a tumor response at the first tumor evaluation [110]. Of the patients studied, 14 had BM, 12 of whom showed intracranial tumor control, 7 had reduced BM and 4 patients had complete remission.

Among patients, 98% reported some adverse effects, with 67% reporting grade 3–4 adverse effects. The most common symptom was peripheral edema, followed by nausea, vomiting and elevated serum creatinine. The incidence of serious treatment-related adverse events was 13%, with 11% of patients having to stop treatment. Dose reduction was required in 23% of subjects included. In one patient, pneumonitis leading directly to death may likely have been related to capmatinib treatment.

### Tepotinib

Tepotinib is also a selective MET tyrosine kinase inhibitor, capable of penetrating the BBB [112]. It has demonstrated efficacy in advanced NSCLC with MET exon 14 skipping mutations in the phase 2 VISION clinical trial [113–115]. Patients with MET amplification were not included in this study. The skipping mutation was detected by histology or liquid biopsy. Patients received the investigational agent in a once-daily oral dose of 500 mg.

In the study, the ORR was 44.7% and the median PFS was 8.9 months. The median overall survival was 17.6 months. Response rates and PFS showed no difference whether the patient received first-line or multi-line treatment with tepotinib. The investigational drug also showed efficacy in elderly patients over 80 (ORR: 35.1%, PFS: 8.6 months). Intracranial tumor control was achieved in the majority of subjects with BM.

Treatment-related adverse events occurred in 86.3% of patients on tepotinib, 24.3% experienced grade 3–4 adverse events, and 12.2% experienced serious adverse events. Three cases were fatal, with death resulting from ILD-related respiratory failure or liver failure. The most common adverse events were peripheral edema, followed by nausea, diarrhea, serum creatinine elevation, and hypoalbuminemia.

### HER2 mutant NSCLC treatment

HER2 is a human epidermal growth factor receptor (EGFR/ERBB2) family and is encoded by the ERBB2 gene. The prevalence of HER2 mutations in patients with non-small cell carcinoma is between 1%–4%. HER2 lesions can develop by three mechanisms: HER2 protein overexpression, HER2 amplification and HER2 gene mutation. IHC, FISC and NGS can be used to detect these lesions. Double platinum-based chemotherapy is the first-line treatment of choice and can be complemented with immunotherapy (ESMO IV.B) [116].

In the DESTINY LUNG01 clinical trial, the HER2 antibody-drug conjugate, trastuzumab-deruxtecan treatment efficacy was investigated. In the study, 91 patients with metastatic HER2 mutant NSCLC received second-line trastuzumab-deruxtecan treatment following standard therapy. In the study, PF was 8.2 months (95% CI, 6.0–11.9), while median OS was 17.8 months (95% CI, 13.8–22.1). Treatment-related adverse events included neutropenia and drug-induced ILD, the latter resulting in 2 deaths identified in the study [117].

The DESTINY LUNG02 phase 2 randomized trial also investigated the efficacy and safety of trastuzumab-deruxtecan following platinum-based treatment in the second line, also at two different doses. At both doses (5.4 mg/kg or 6.4 mg/kg every 3 weeks), a significant and sustained antitumor effect with an acceptable safety profile was observed, but the lower dose had a lower rate of drug-induced ILD [118]. The study’s results led to EMA approval of second-line trastuzumab-deruxtecan treatment for NSCLC with metastatic or unresectable HER2 mutations. First line trial phase 3 is recruiting [119].

### Treatment of NTRK gene fusion-positive NSCLC

Neurotrophic tyrosine receptor kinase (NTRK) gene fusions initiate downstream signaling pathways, such as the AKT and MEK pathways, and are present at a very low frequency (<1) in solid tumors [1].

Entrectinib is an NTRK and ROS1 inhibitor that can penetrate the central nervous system. In a clinical study of the efficacy of entrectinib in a total of 22 NSCLC patients, the mPFS was 14.9 months (95% Cl, 6.5–30.4), median OS results are not yet available [120].

Larotrectinib is a sensitive tropomyosin receptor kinase inhibitor. In two multicentre clinical trials, a total of 20 NRTK gene fusion-positive patients were tested for the efficacy of larotrectinib. Median PFS outcome was 35.4 months (95% CI, 5.3–35.4), and the median OS was 40.7 months [121].

## Discussion

Patients with guideline-recommended molecular alteration-based therapies have better outcomes with first-line targeted therapy for advanced-stage NSCLC [1]. In a retrospective study, others showed a significant increase in OS in patients with non-squamous NSCLC with molecular testing available compared to non-tested patients [122]. Importantly, comprehensive NGS vs. incomplete or no testing before initiating first-line therapy impacts the OS (22.1 vs. 11.6 months, *p* = 0.017) respectively [123]. Nevertheless, a multidisciplinary approach is essential in finding the proper diagnostic procedures and treatments to personalize NSCLC therapy. There is a broad repertoire of targeted therapies in the standard of care settings. However, there is a need for improvements; therefore, participation in clinical trials is especially encouraged [2]. Accurate imaging-based clinical staging and tissue availability influence subsequent molecular assay-based personalized therapeutic decisions in multidisciplinary teams (MDT) before first line therapy administration. The gold standard for molecular testing in NSCLC is tissue-based testing. Liquid biopsy-based ctDNA detection can guide therapy; however, it should not be used instead of tissue samples. However, the plasma-first approach is recommended if tissue is unavailable [124]. Molecular testing for stage IV NSCLC with reflex testing is associated with shorter turnaround times. There is an emerging requirement for testing in early-stage disease. Formalin-fixed paraffin-embedded (FFPE) material suits for most molecular analyses and non-acid decalcification approaches on bone biopsies. Molecular assays, such as cell blocks, direct smears, or touch preparations, are recommended in order not to miss a targetable genetic alteration. Nevertheless, adequate biopsy sampling should ensure that the sample is suitable for molecular analysis, and in small specimens, minimal IHC should be used to preserve the tissue for molecular studies [2]. Accordingly, the acceptable terms NSCLC favor adenocarcinoma or favor squamous cell carcinoma is recommended with any extent of adenocarcinoma component in a biopsy specimen that is otherwise squamous should trigger molecular testing [2]. At a minimum, EGFR and ALK testing is recommended before initiating immunotherapy because rapid and sensitive tests are available [2, 124]. MDT is best assisted by complete-scale molecular testing for a first-line treatment decision that includes NGS and PDL1 expression [123]. DNA-based NGS oncology panels are recommended to detect EGFR, KRAS, MET, RET, HER2, and BRAF. ALK, ROS1, and NTRK1/2/3 alterations can be identified with FISH. IHC, for screening purposes with low specificity, can also be applied. Therefore, validation with NGS DNA panels with reasonable specificity may detect ALK, RET, and NTRK2 but may underdetect ROS1, NTRK1, and NTRK3 fusions. In the case of RET and METex14, skipping events, RNA-based NGS is preferable to DNA-based NGS or fusion detection [2].

Following the expansion in molecular alteration-based targeted therapy in advanced stages, recently, attention has turned to early-stage cases and resection specimens. Recent advancements in the NSCLC adjuvant treatment setting, the molecular diagnostics for EGFR and ALK in the early stage, indeed necessary to exclude targetable alterations to pave the way to proceed with immunotherapy based on PD-L1 expressors. Accordingly, molecular testing of early-stage resectable NSCLC before neoadjuvant nivolumab plus chemotherapy was performed in CheckMate 816 [125]. Additionally, molecular testing was performed to exclude driver oncogenes in the perioperative early-stage setting in the AEGEAN study on durvalumab plus neoadjuvant chemotherapy [126].

Osimertinib is the first approved targeted therapy based on the ADAURA trial that enrolled patients with classical EGFR mutant (ex19del/L858R) after complete resection, stage I/B -III/A [127, 128]. Adjuvant ALK therapy is currently in a clinical trial [129]. According to an interim analysis of the ALINA trial, adjuvant targeted treatment with alectinib was associated with significant disease-free survival (DFS) benefits compared with platinum-based chemotherapy, with favorable results for alectinib seen in both the stage II–IIIA population (n = 231; hazard ratio [HR] 0.24; 95% confidence interval [CI] 0.13–0.45; *p* < 0.0001) and the intention-to-treat (ITT) (stage IB–IIIA) population (n = 257; HR 0.24; 95% CI 0.13–0.43; *p* < 0.0001) [129, 130].

**FIGURE 5:**
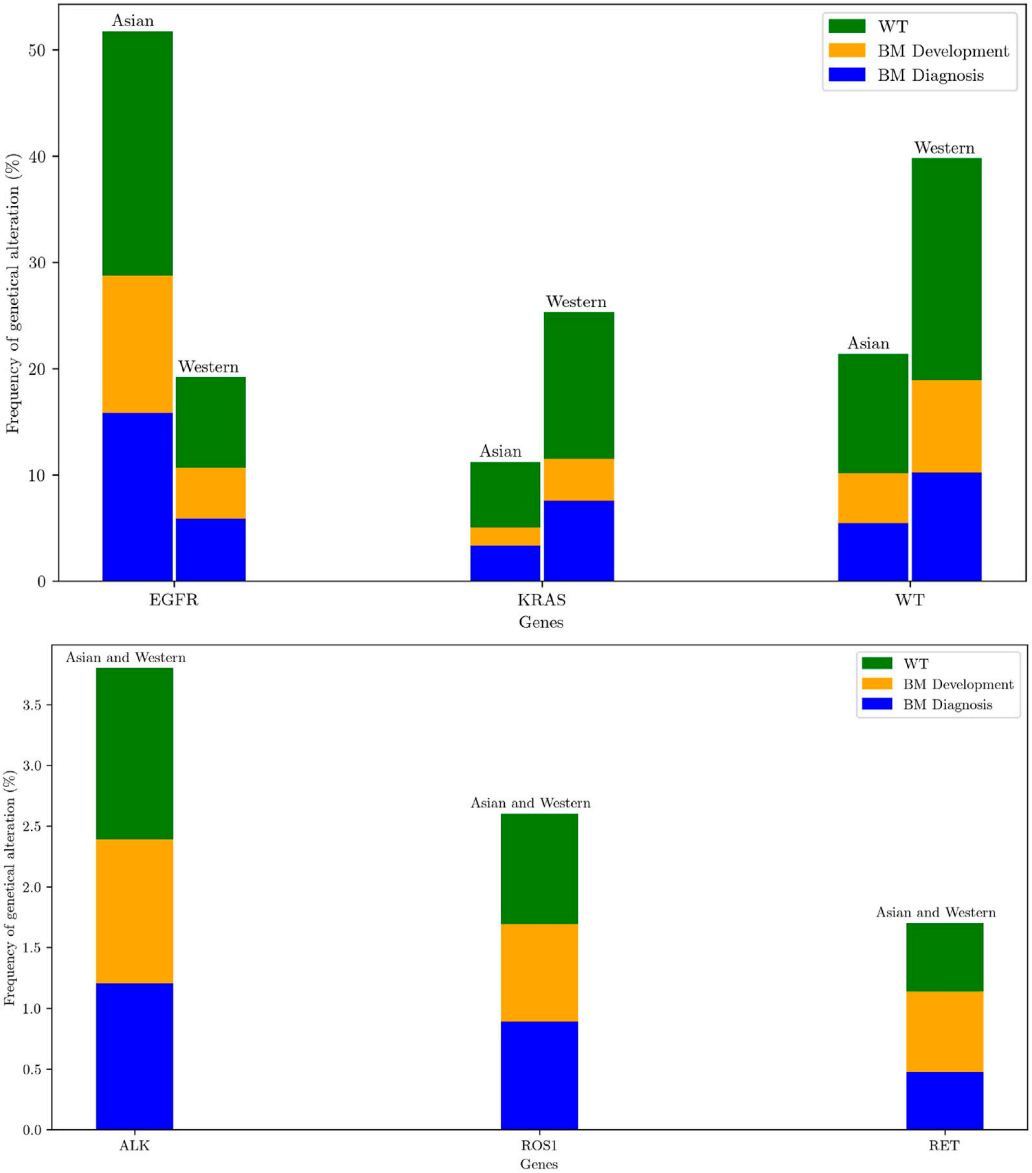
Frequency of oncogenic drivers for East-Asian and Western populations [134]. Bars are stratified according to proportions of brain metastases (BM) at diagnosis (blue) and BM development after diagnosis (orange) and pan-wild-type for all genetic alterations reported including rare ones (WT, green) [131]. For visual enhancement low frequencies of ALK, ROS1, RET genetical alterations are presented in a separate figure.

A key factor with targeted therapies includes the control rate of BM; however, there is a significant difference between targeted therapies regarding brain efficacy. New-generation targeted therapies with blood barrier penetration increased the prognosis of brain metastatic NSCLC patients [58]. 20%–30% with advanced NSCLC were found to have BM at diagnosis [131, 132]. Figure 5 shows the distribution of BM according to genetical alterations. A recent meta-analysis suggests that patients with ALK-positive and EGFR-positive NSCLC had higher rates of BM development than other genomic alterations and wild-type tumors [131]. Others showed an association with metastasis development in tumors with ROS1, MET, and RET alterations [131]. However, a meta-analysis does not support a higher rate of BM in these cases compared with wild-type cohorts [131]. BM are frequent in advanced EGFR-mutated or ALK-rearranged NSCLCs, with an estimated >45% of patients with CNS involvement by 3 years of survival with targeted therapies [133].

The intracranial tumor response to TKIs is shown in Table 1 [135]. Patients with EGFR, ALK, and ROS1 positive tumors with oligo- or asymptomatic BM should be treated by upfront systemic targeted therapy [ESMO: III, B] [136]. Of note close MRI surveillance is strongly recommended [1]. The upfront use of radiotherapy might be considered upon BM progression [137]. However, there is no available data on trials comparing the two strategies to assess the impact of delayed radiation in terms of survival or neurologic deficit [138]. ALK inhibitors with CNS activity include Brigatinib, Lorlatinib, and Alectinib [43, 45, 46]. ROS1: Entrectinib is recommended in patients with BM [ ESCAT: I-B]. Compared with earlier-generation drugs, CNS activity of the EGFR TKI, osimertinib showed better intracranial response rates, including stable CNS metastatic cases, in 60% [23, 139].

**TABLE 1:** Intracranial objective response rate in patients with BM according to the presence of drive oncogenes and targeted therapy administration [135].

Study	Year/Trial ID	Driver oncogene	Targeted therapy	Intracranial efficacy (%)
FLAURA	2014 NCT02296125	EGFR Ex19del, Ex21 L858R	osimertinib	91
ALEX	2014 NCT02075840	ALK	alectinib	81
ALTA-1L	2016 NCT02737501	ALK	brigatinib	78
CROWN	2017 NCT03052608	ALK	lorlatinib	82
STARTRK2	2015 NCT02568267	ROS1	entrectinib	79
Geometry Mono-1	2015 NCT02414139	MET exon 14 skipping mutation, MET amplification	capmatinib	54
VISION	2016 NCT02864992	MET exon 14 skipping mutation, MET amplification	tepotinib	55
LIBRETTO-001	2017 NCT03157128	RET	selpercatinib	82
ARROW	2017 NCT03037385	RET	pralsetinib	78
CodeBreaK 100	2018 NCT03600883	*KRAS* G12C	sotorasib	25

## Conclusion

Recent expansion in the targeted treatment options into the adjuvant setting of non-small cell lung cancer using accurate pathology diagnostics can minimize the number of excluded patients from molecular diagnostics. Accordingly, careful planning of subsequent hierarchical steps of diagnostic and therapeutic aspects can lead to improved outcomes without excluding patients from best-match targeted therapy. The selection of biopsy procedures and sites, tissue processing, and interpretation, followed by accurate molecular testing-based biomarker identification, is critical. Accordingly, the complexity of theranostics and possible resistance mechanisms can lead to better quality of life and outcomes in special populations, in patients with BM. Future trials should address drug properties such as CNS activity and other special populations, including oligometastatic disease and the emergence of resistance genes to maximize patient survival. Despite the novel standard of care therapies, clinical trials are guideline-recommended options to improve patient outcomes. Accordingly, the therapeutic options are expanding based on the innovative and positive trial results.
